# Self-Driven Photo-Polarized Water Molecule-Triggered Graphene-Based Photodetector

**DOI:** 10.34133/research.0202

**Published:** 2023-07-31

**Authors:** Shisheng Lin, Chang Liu, Xin Chen, Yi Zhang, Hongtao Lin, Xutao Yu, Yujiao Bo, Yanghua Lu

**Affiliations:** ^1^ College of Information Science and Electronic Engineering, Zhejiang University, Hangzhou 310027, P. R. China.; ^2^ Hangzhou Gelanfeng Technology Co. Ltd, Hangzhou 310051, P. R. China.; ^3^ State Key Laboratory of Modern Optical Instrumentation, Zhejiang University, Hangzhou 310027, P. R. China.; ^4^ Key Laboratory of Wide Bandgap Semiconductor Materials and Devices, HCSemitek Corporation, Yiwu 322009, P. R. China.; ^5^ Smart Materials for Architecture Research Lab, Innovation Center of Yangtze River Delta, Zhejiang University, Jiaxing 314100, P. R. China.

## Abstract

Flowing water can be used as an energy source for generators, providing a major part of the energy for daily life. However, water is rarely used for information or electronic devices. Herein, we present the feasibility of a polarized liquid-triggered photodetector in which polarized water is sandwiched between graphene and a semiconductor. Due to the polarization and depolarization processes of water molecules driven by photogenerated carriers, a photo-sensitive current can be repeatedly produced, resulting in a high-performance photodetector. The response wavelength of the photodetector can be fine-tuned as a result of the free choice of semiconductors as there is no requirement of lattice match between graphene and the semiconductors. Under zero voltage bias, the responsivity and specific detectivity of Gr/NaCl (0.5 M)W/N-GaN reach values of 130.7 mA/W and 2.3 × 10^9^ Jones under 350 nm illumination, respectively. Meanwhile, using a polar liquid photodetector can successfully read the photoplethysmography signals to produce accurate oxygen blood saturation and heart rate. Compared with the commercial pulse oximetry sensor, the average errors of oxygen saturation and heart rate in the designed photoplethysmography sensor are ~1.9% and ~2.1%, respectively. This study reveals that water can be used as a high-performance photodetector in informative industries.

## Introduction

In the traditional PN junction, the Fermi level difference between P-type and N-type semiconductors creates a built-in electric field that can block originally diffused carriers and reach equilibrium. These PN junctions can be used as photodetectors in which the photogenerated carriers of incident light excitation are separated by a built-in electric field to output electrical signals [1–3]. Gallium nitride (GaN) as a representative wide bandgap has been widely used for semiconductor PN junction photodetectors and light-emitting diodes owing to its superior chemical stability and radiation hardness [4,5]. Graphene (Gr) was created as a novel 2-dimensional semiconductor with carrier multiplication and superconductivity due to its strong electron–electron interactions and because it is bandgapless [6–9]. Combining 2D materials and 3D semiconductors to form heterojunctions, Gr/GaN heterojunction has been widely reported for optoelectronic devices based on a traditionally PN junction [10–12].

Water (W), which is widely distributed on earth and constitutes more than 70% of the composition of the human body, is recognized as the source of life and composed of 2 hydrogen atoms and one oxygen atom. Water has gained wide attention as a creative lightweight in situ energy, which can be applied to the field of Internet of Things (IoT) [13–15]. Electrolytes can interact with water molecules in many ways, which is possible because water molecules are strongly responsive to electrostatic fields in simple ions and charged macro molecules. In our previous work [16,17], the energy of low-energy, disordered water flow converted into continuous direct current (DC) has been demonstrated via introducing polarized water molecules as mobile dielectrics in dynamic diodes. However, the effect of photogenerated carriers in the polarization process has been neglected. Combining with the photoelectric effect, aqueous electrolyte solutions can be used to construct solid–liquid photodetectors.

In this work, we uncover the mix-phased high-performance ultraviolet (UV) photodetector, in which inserting polar liquid into the PN junction can generate persistent photo-polarized current under illumination. Under the action of the polar liquid chemical potential and the semiconductor Fermi level, the photogenerated electrons and holes will continuously move to the opposite sides of the polar liquid. Under zero bias voltage, The Gr/NaCl (0.5 M)/W/N-GaN device exhibits a responsivity (*R*) and detectivity (D^*^) up to 130.7 mA/W and 2.3 × 10^9^ Jones with 350 nm, respectively. With the replacing wavelength-selective materials, the Gr/NaCl (0.5 M)W/N-GaAs device exhibits good photoelectric conversion performance in the visible (Vis) and near-infrared (NIR) regions. Immediately, the photoplethysmography (PPG) waveforms were read and processed under the irradiation of 660-nm and 850-nm light-emitting diodes (LEDs); the obtained heart rate (HR) and oxygen blood saturation (SpO_2_) are 69.7 to 74.2 beats per minute and 93.8% to 95.6%, respectively. Our method reveals the polarizing properties of polar liquids in the photoelectric effect, bringing a novel and promising approach for converting input light energy into sustained polarized electricity and providing an additional dimension into photodetection and health monitoring.

## Results and Discussion

**Fig. 1.**
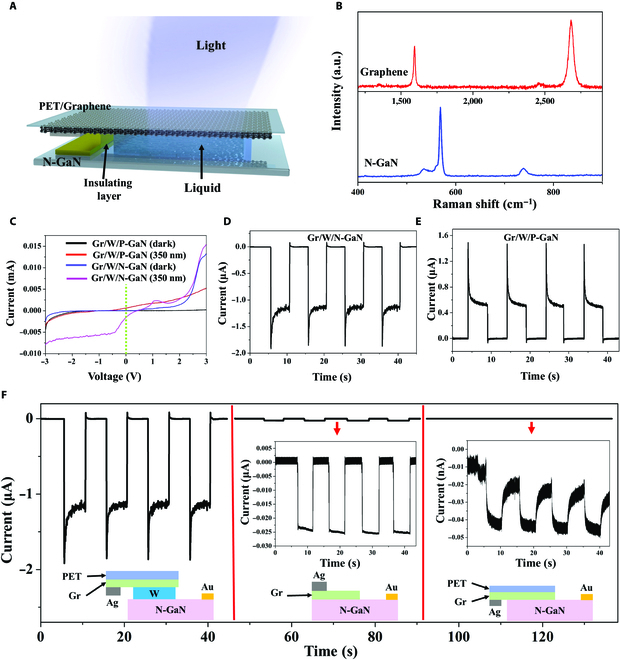
Device design and results of the Gr-based photodetector. (A) Schematic diagrams of device structure. (B) Raman spectrum of bi-layer graphene on Si/SiO_2_ substrate and N-GaN, respectively. (C) *I*–*V* curves of the Gr/W/N-GaN and Gr/W/P-GaN photodetector under dark and 350 nm illumination. The photo-polarized current of (D) Gr/W/N-GaN and (E) Gr/W/P-GaN under 350 nm illumination with 261 μW/cm^2^. (F) The photo-polarized current of different device structures under the same test conditions. The red arrow points to the enlarged view of photo-polarized current.

**Fig. 2.**
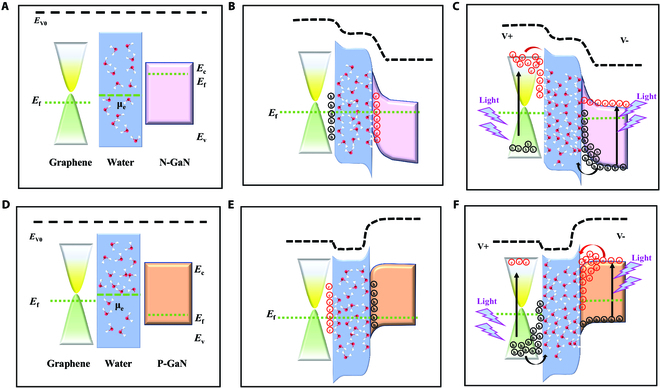
Discussion based on Fermi level and molecular polarization principles. The band structure physical mechanisms of the Gr/W/N-GaN and Gr/W/P-GaN photodetector. (A and D) Before contact, (B and E) after contact, and (C and F) under light illumination.

A 3-dimensional (3D) schematic model of the Gr/liquid/N-GaN photodetector is shown in Fig. 1A, where the polar liquid is sandwiched between a bi-layer Gr, N-GaN, and an insulating layer. The practical image of the Gr/liquid/N-GaN photodetector is shown in Fig. S1A, and the corresponding dimensions are marked. The effective illumination area of the Gr/liquid/N-GaN photodetector is 0.15 cm^2^ and overall device size is 1.65 cm^2^. In order to more clearly describe the dimensions of each part, the disassembled 3D view of the Gr/W/N-GaN device is shown in Fig. S1B. To confirm the structural properties of related semiconductors, the typical Raman spectroscopy is recorded in Fig. 1B; the bottom panel shows that the obvious characteristic positioned peaks at 568.53 and 738.33 cm^−1^ correspond to the E_2_ (high) and A_1_ (LO) phonon modes of GaN, and the peak located at 534.42 cm^−1^ corresponds to the A_1_ (TO) phonon mode [12]. In the top panel, the Raman peaks observed at 1,592.3 and 2,685 cm^−1^ can be attributed to the G peak and 2D peak of Gr [18,19]. Remarkably, the extremely weak D peak (1,345 cm^−1^) reveals high quality and limited defects of graphene [20]. The current–voltage (*I*–*V*) curves of the Gr/W/N-GaN and Gr/W/P-GaN under dark and 350 nm illumination are illustrated in Fig. 1C, where 2 devices show rectification behavior in opposite directions. The current–time (*I*–*T*) curve of the Gr/W/N-GaN photodetector under zero bias voltage with multiple cycles of light switching is shown in Fig. 1D. Obviously, a spike current belonging to the transient polarization process appears at the moment the light is turned on; subsequently, photo-polarized current tends to be steady state. A smaller reverse spike appears after turning off the light, which belongs to the depolarization process. The transient and steady-state photo-polarized current of Gr/W/N-GaN basically reached 1.8 μA and 1.1 μA, respectively. The *I*–*T* curve for Gr/W/P-GaN is shown in Fig. 1E and the device demonstrates reliable switching repeatability. Under the same measurement conditions, the transient and steady-state photo-polarized current of Gr/W/P-GaN basically reached 1.4 μA and 0.5 μA, which is smaller than Gr/W/N-GaN. Specifically, the polarization current direction of Gr/W/P-GaN is opposite to Gr/W/N-GaN, which is related to the energy band structure of the device, and a detailed analysis is given in Fig. 2.

The control experiments directly show the advantages of inserting polar liquids between the Gr and GaN in constructing high-performance self-driven photodetectors. As shown in Fig. 1F, compared with the traditional solid-state Gr/N-GaN heterojunction photodetector (middle panel) and the direct physical contact Gr/N-GaN structure without water (right panel), the Gr/W/N-GaN photodetector (left panel) exhibits a good performance. This type of photodetector could have a good performance that is competitive with the traditional PN diode-based photodetector. Notably, in conventional solid-state Gr/N-GaN heterojunction photodetectors, after being excited by incident light, photogenerated carriers are separated instantly by the built-in electric field. Therefore, transient photo-polarized current will not occur.

Notably, Gr/W/N-GaN and Gr/W/P-GaN exhibit different directional photocurrent outputs under the same conditions. We explain the emergence of bipolar photocurrents from band structures’ physical mechanisms. The Fermi level of Gr after wet transfer is determined by *S* = |Δ*E*_f_| × 42 cm^−1^eV^−1^ [21], where *S* is the difference between the G peak relative to 1,580 cm^−1^, and Δ*E*_f_ is the difference between the Gr Fermi level relative to the Dirac point. In theory, the Dirac point in graphene is around 4.6 eV [22]. The G peak can be obtained from the Raman spectrum at 1,592.3 cm^−1^; therefore, the work function of Gr is evaluated around 4.89 eV. Meanwhile, the work functions of N-GaN and P-GaN are around ~4.0 eV and ~6.1 eV due to the doping Si and Mg [23,24]. The work function of water can be estimated to 4.2 eV under standard atmospheric [25]. As indicated in Fig. 2A and D, before contact, the water molecules are disordered state. After contact (Fig. 2B and E), owing to the work function difference, energy bandgap alignment occurs between GaN and Gr. Meanwhile, the water molecules at the solid–liquid interface will be ordered polarized due to the Fermi level difference. As illustrated in Fig. 2C and F, under the excitation of light, the photogenerated carriers drift to the interface in large quantities, which polarizes many water molecules. As shown in Fig. 2C, the holes in N-GaN will accumulate at the interface between W/N-GaN, and the corresponding electrons in Gr will accumulate at the interface between Gr/water. Compared with Gr/W/N-GaN, the carrier types at the interface of Gr/W/P-GaN device are completely opposite. Meanwhile, the voltage–time (*V*–*T*) curve of Gr/W/N-GaN photodetector with different parallel resistances is shown in Fig. S2. Obviously, the photogenerated voltage is good following light being switched on and off, and rises with increasing shunt resistance. We further discuss the relationship between the number of graphene layers and the polarization current. The polarization photocurrent of the bi-layer graphene was slightly larger than that of the other samples (Fig. S3). The photo-current is dependent on the balance between optical absorption ability and resisitivity of graphene as a function of layer numbers. As thick graphene layers reduce the resisitivity while increases the absorptions, there is the optimized layer number of graphene for the photodetector, herein, bilayer graphene has the best performance. We speculate that the photocurrent is probably related to optical ability absorption, and the carriers’ recombination in multilayer graphene makes it difficult to polarize more polar molecules to participate in charge transport.

**Fig. 3.**
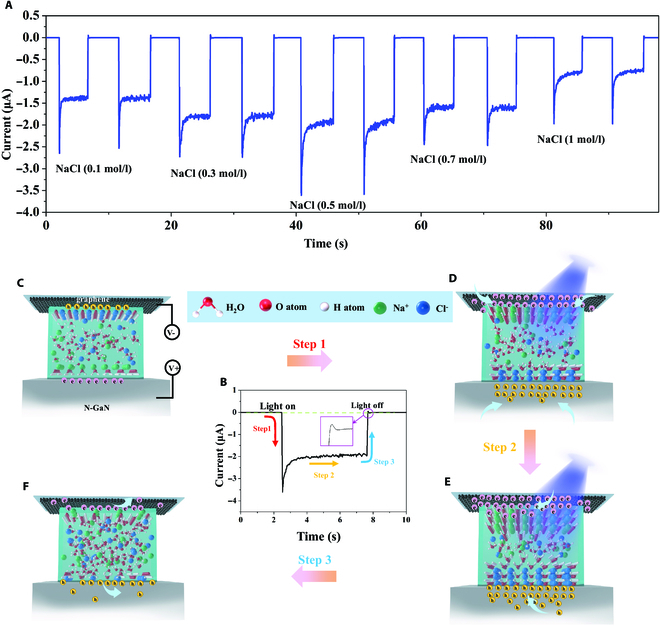
The physical mechanism of the polarized molecule-triggered photodetector. (A) The photo-polarized current of Gr/NaCl (aq)/N-GaN with different concentrations of NaCl (0.1 to 1 M, step with 0.2 M) under 350 nm illumination. Working mechanism of the Gr/NaCl (0.5 M)W/N-GaN photodetector under light illumination. (B) *I*–*T* curves of photodetector. (C) Equilibrium state of the Gr/NaCl (0.5 M)W/N-GaN photodetector after the contact. (D) Photogenerated carrier accumulation induced the polarization state after the semiconductor was excited. (E) Continuously stable polarization state induced by accumulation under stable illumination. (F) Depolarization process after turning off the light.

Molecular polarization and photogenerated carriers coupling in polar liquids can efficiently generate photocurrents. In order to further confirm that the photocurrent originates from the polarization process, we use the non-polar liquid n-hexane as a medium to insert between Gr and N-GaN. The *I*–*T* curve of the Gr/n-hexane/N-GaN device is shown in Fig. S4. Clearly, this device does not generate photocurrent during the optical switching process, confirming that the photocurrent is caused by the liquid polarization and photogenerated carriers coupling. Furthermore, we replaced the polar liquid with a salt solution of polar liquid, and the photocurrent has been further improved. As indicated by Fig. 3A, after replacing water with NaCl solution, the photo-polarized current is markedly improved under 350 nm illumination. The transient photo-polarized current of Gr/NaCl (aq)/N-GaN reached 2.4 μA, 2.7 μA, 3.6 μA, 2.4 μA, and 1.9 μA under 350 nm irradiation, corresponding to Gr/NaCl (0.1 M)W/N-GaN, Gr/NaCl (0.3 M)W/N-GaN, Gr/NaCl (0.5 M)W/N-GaN, Gr/NaCl (0.7 M)W/N-GaN, and Gr/NaCl (1.0 M)W/N-GaN samples, respectively. Compared with the Gr/W/N-GaN, the salt ions increase the conductivity of the water and also promote the interaction and ordered alignment of the water molecules [26–28], thus increasing the performance of the photodetector and inducing maximum responsivity, which increased by 51%. Meanwhile, we find that the transient polarization current decreases obviously when the concentration of NaCl solution exceeds 0.5 M, excessive ions will accumulate to the solid–liquid interface prior to the polarization of water molecules, and the electric field shielding weakens the steady-state polarization photocurrent. In addition, we speculate that sodium ions will form sodium ion hydrates with water molecules [29], and the oxygen atoms in the water molecules will point to the nano ions, thus inhibiting the occurrence of polarization.

To further exhibit the polarization mechanism more intuitively, we show the one switching cycle of Gr/NaCl (0.5M)W/N-GaN photodetector in Fig. 3B and the working mechanism in Fig. 3C to F. After contact, different Fermi levels between materials induce polarization of water molecules at the interface, where oxygen atoms point to graphene and hydrogen atoms point to N-GaN [16]. The Na^+^ and Cl^−^ in the solution are attracted by different polarity charges and move to opposite directions (Fig. 3C). As shown by step 1, the semiconductor is instantly excited by the irradiation, and a large number of photogenerated carriers will move to the interface between the semiconductor and the liquid under the action of the built-in field, inducing the rapid accumulation of positive and negative ions in the salt solution and molecular polarization at the interface. The movement of the carriers will generate a higher photo-polarized current, as indicated by the process of step 1 in Fig. 3B. In the next step (Fig. 3D), the polarization current tends to be stable with more water molecules being polarized and ions move to the solid–liquid interface, as indicated by the process of step 2 in Fig. 3B. As shown in step 3 of Fig. 3B, after the light source is turned off, since the photo-induced carriers instantaneously relax and recombine with the lattice defects immediately, most of the water molecules are depolarized and result in a negative current output. The physical map changes from Fig. 3E and F.

**Fig. 4.**
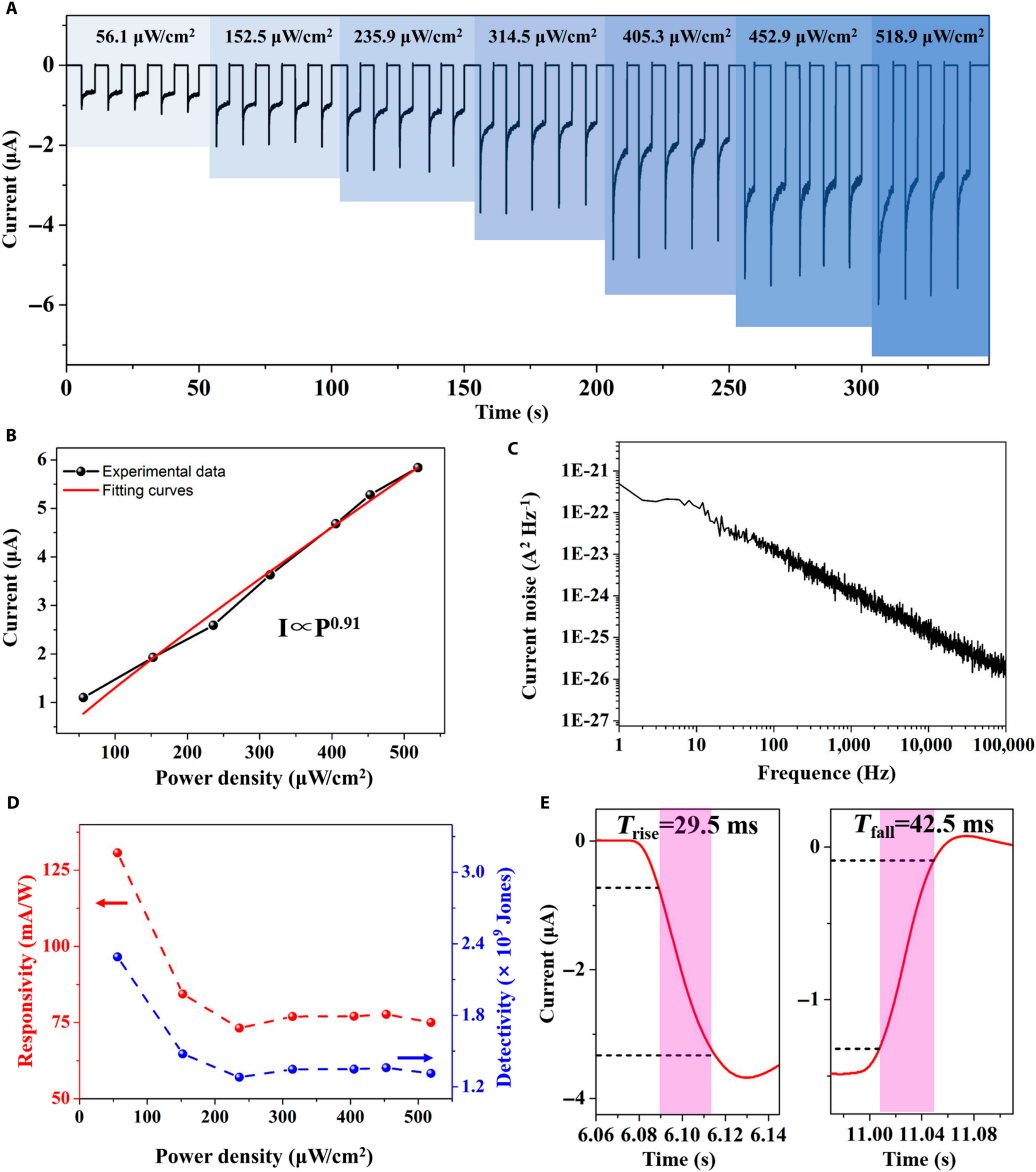
The properties of the Gr/NaCl (0.5 M)W/N-GaN photodetector. (A) The photo-polarized current of the Gr/NaCl (0.5 M)W/N-GaN photodetector under 350 nm with different optical power intensities. (B) Fitting of the linear relationship between extracted transient photo-polarized current and optical power as a function of optical power. (C) 1/*f* noise spectra of the Gr/NaCl (0.5 M)W/N-GaN photodetector under zero bias voltage. (D) Responsivity and detectivity of transient photo-polarized current as a function of incident optical power intensity under zero bias. (E) Response time and recovery time of Gr/NaCl (0.5 M)W/N-GaN with 350 nm illumination.

The self-powered *I*–*T* curves of the Gr/NaCl (0.5 M)/W/N-GaN photodetector under 350 nm with different optical intensities of 66.1, 152.5, 235.9, 314.5, 405.3, 452.9, and 518.9 μW/cm^2^ are shown in Fig. 4A, respectively. The transient/steady-state photo-polarized current increases with increasing optical power density, which is due to the fact that more photo-induced charge carriers are coupled to water molecules and ions under higher intensities of light. It is worth noting that the polarized current increases gradually without a saturation, which means that the prepared mis-phase photodetector could have a good performance that is competitive with the traditional solid-state photodetector. The relationship between the transient polarization current and the incident optical power density is shown in Fig. 4B, and the dependence of the photo-polarized current on the optical power could be fitted by a nonlinear power law: *I =A×P^α^*, where *A* is the scaling factor and the exponent *α* is the linearity of the photo-polarized current on the power intensity. The fitting consequence reveals that the acquired *α* is approximately 0.91 in the range of 0 to 500 μW/cm^2^, showing a good linear behavior. The *V*–*T* curves of the Gr/NaCl (0.5 M)W/N-GaN under 350 nm with different optical intensities are shown in Fig. S5A. The device is connected in parallel with 220-kΩ resistors and exhibits excellent stability and light power dependence. The extracted peaks of the corresponding transient photo voltage are shown in Fig. S5B, where the photo voltage gradually reaches saturation with the light power increasing. The response voltage barely increases after reaching the saturated 0.021 V when the optical power is greater than 60 μW/cm^2^. When the light power is in the range of 0 to 60 μW/cm^2^, the response voltage of the device and the incident light power satisfy a linear relationship.

To further evaluate the overall performance of the photodetector, *R* and D^*^ are indispensable parameters, representing the ability to convert unit optical power into photo-current and detect weak signal. *R* can be determined by Eq. 1:

$$R=\frac{I_{L}-I_{D}}{\mathrm{PS}}$$ (1)

where *I_L_* is the transient/steady-state photo-polarized current, *I_D_* is the dark current, and *P* is the incident optical power density. *S* is the effective illumination area of Gr/NaCl (aq)/N-GaN. Meanwhile, D^*^ can be determined by Eq. 2 [30]:

$$D^{*}=\frac{\sqrt{\mathrm{SB}}}{\mathrm{NEP}}=\frac{R\sqrt{S}}{S_{n}}$$ (2)

where *S* represents the active work area of the detector. *B* represents measurement bandwidth (1 Hz). *NEP* represents the noise equivalent power. *S_n_* represents the dark current noise. Generally, the total dark current noise of the device mainly consists of the flicker noise (1/*f* noise), shot noise, and thermal noise [31]. 1/*f* noise spectral density was recorded as shown in Fig. 4C, and the noise current is linear with frequency with a slope of 1/*f*, proving that our photodetector has a typical 1/*f* noise characteristic. Under zero applied voltage, the obtained 1/*f* noise is 4.9 × 10^−22^ A^2^·Hz^−1^ at a frequency of 1 Hz. Moreover, the shot noise (*N_s_*) is induced by dark current and determined by the following formula [32] (Eq. 3):

$$N_{s}=2qI_{D}\Delta f$$ (3)

where *q* and Δ*f* represent the charge constant and bandwidth, respectively. The thermal noise (*N_t_*) can be determined by the following formula [2] (Eq. 4):

$$N_{t}=\frac{4\mathrm{kTB}}{R_{s}}$$ (4)

where *k*, *T*, and *R_s_* represent the Boltzmann constant, the experimental temperature (about 300 K in this experiment), and the resistance of the Gr/NaCl (aq)/N-GaN device, respectively. The *N_s_* and *N_t_* are calculated to be 1.1 × 10^−27^ A^2^·Hz^−1^ and 2.9 × 10^−26^ A^2^·Hz^−1^, respectively. Therefore, 1/*f* noise is the dominant noise of the device under low frequency and D* is mainly decided by 1/*f* noise.

Figure 4D shows the *R* and D^*^ of transient photo-polarized current for the Gr/NaCl (0.5 M)W/N-GaN photodetector as a function of incident light power density. The maximum *R* and D^*^ reaches values of 130.7 mA/W and 2.3 × 10^9^ Jones under zero bias voltage. The nonlinear relationship between *R* and light power density is probably caused by the light power density affecting the trap density of state occupancy in GaN and graphene. In addition, the higher power density leads to a gradual saturation of the carrier generation rate [33,34]. The *R* and D^*^ of steady-state photo-polarized current for the Gr/NaCl (0.5 M)W/N-GaN photodetector as a function of incident light power density are given in Fig. S6. The maximum *R* and D^*^ reach values of 87.9 mA/W and 1.6 × 10^9^ Jones under zero bias voltage, respectively. There is a delay between the opening and closing of a photodetector. The response time is the most direct physical quantity to measure the difference between the received optical signal and the output electrical signal of the detector. The time between 10% and 90% of the maximum value of the current after turning on the light source is usually called the rise time (*t*_rise_) and the time between 90% and 10% of the maximum value of the current after turning off the light source is called the fall time (*t*_fall_). An enlarged plot of the response of one switching cycle is shown in Fig. 4E. The corresponding *t*_rise_ and *t*_fall_ are 29.5 ms and 42.5 ms, respectively. Furthermore, the relationship between the liquid thickness and the photo-polarized current was investigated. As shown in Fig. S7, the photo-polarized current decreases with increasing distance between PN junctions, which may be due to the thicker liquid thickness weakening the polarization intensity of the semiconductor to the whole liquid. Besides, the reproducible dynamic response of the device by switching the light on/off in frequency of 1 Hz is shown in Fig. S8. The optical switching behavior and photo-polarized current have no degradation after thousands of cycles, demonstrating the good stability and repeatability of this device. Scanning electron microscopy (SEM) images of PET/graphene before and after the light on/off cycle test are provided in Fig. S9A and B. The bi-layer graphene was uniformly covered on the PET support layer, and the folds shown on the surface are due to the PET substrate. There is no significant change after repeated measurements. Furthermore, the optical photos and optical microscopy images of the bi-layer graphene (0.8 cm × 1 cm) transferred to SiO_2_/Si substrates are shown in Fig. S9C and D. The large-area graphene exhibits good optically uniform, without obvious folds and cracks.

**Fig. 5.**
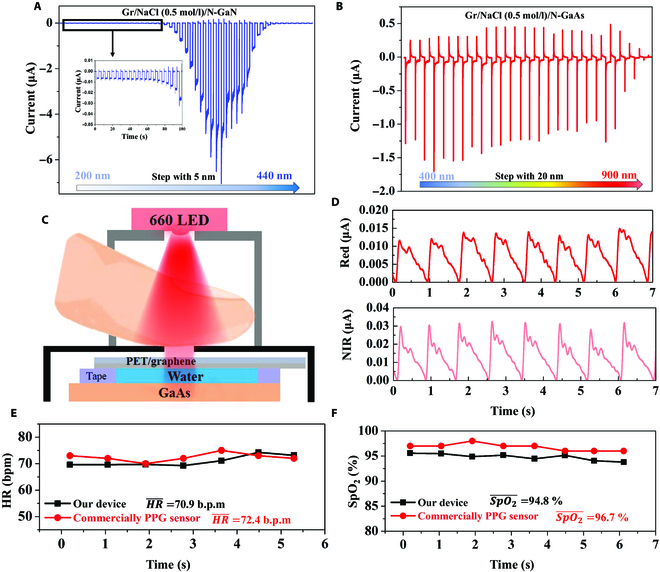
Visible and NIR photodetector fabrication and PPG testing. (A and B) Time-resolved photo-polarized current of the (A) Gr/NaCl (0.5 M)W/N-GaN photodetector under different wavelengths (200 nm to 440 nm, step with 5 nm) and the (B) Gr/NaCl (0.5 M)W/N-GaAs photodetector under different wavelengths (400 nm to 900 nm, step with 20 nm). (C) Schematic diagram of the PPG measurement. (D) Processed AC signals obtained from the red light and NIR light. Calculated (E) HR and (F) SpO_2_ based on our device and the commercial PPG sensor. bpm, beats per minute.

Figure 5A reveals the curve of photo-polarized current as a function of incident wavelength. The corresponding power density of different wavelength is shown in Fig. S10b. The photo-polarized current increases with the increase of the incident wavelength and reaches the maximum at 355 nm, which is related to the absorption capacity of the device. There is also a corresponding photo-polarized current response in the solar blind region; however, photogenerated carriers excited by larger photon energies at deeper positions within the semiconductor will recombine rapidly. The UV–Vis absorption spectra of the N-GaN exhibits a steep absorption edge around 360 nm (Fig. S11), which corresponds to its bandgap, and has negligible absorption in the Vis region. The typical spectral responses of Gr/NaCl (0.5 M)W/N-GaN photodetector are exhibited in Fig. S10a. The UV–Vis rejection ratio defined as the ratio of the responsivity at 330 and 400 nm is about 1.4 × 10^2^. This work provides the potential way to break through the constraints on the lattice match of heterojunction semiconductor photodetector, where we could freely select the appropriate semiconductor in combination with a polar liquid depending on the wavelength that needs to be detected. Integrating GaAs with broadband absorption into the device for photoelectric conversion measurement is shown in Fig. 5B. The device exhibits significant transient/steady-state photo-polarized current currents in the Vis and NIR regions and decreases sharply after 880 nm, and the corresponding *R* and optical power density are given in Fig. S10C and D.

PPG sensors are part of an important field of application for photodetectors and commonly used in medical devices for health monitoring [35,36]. Since the absorption coefficients of oxyhemoglobin (HbO_2_) and deoxyhemoglobin (Hb) are markedly different in the red and NIR regions, the PPG method can be used for non-invasive physiological health monitoring [37]. Herein, the Gr/NaCl (0.5 M)W/N-GaAs photodetector has a responsiveness at both red and NIR wavelengths and could be integrated as a receiver in a noninvasive pulsed blood oxygen measurement sensor system, and the operation details are provided in Materials and Methods. The schematic diagram and photograph of the blood oxygen-monitoring device are shown in Fig. 5C and Fig. S12, respectively. The PPG signals can be divided into 2 components. The DC component is mainly derived from the absorption of light by the detector through muscles, bones, veins, and other connected tissues, and this part of the light absorption is essentially constant. The alternate current (AC) component is mainly derived from the absorption of light by the detector through the blood flowing in the arteries, which is a direct reflection of the change in vessel diameter. The characteristics of blood flow can be obtained by extracting AC signal from PPG. The AC signal from the 660-nm and 850-nm incident light are shown in Fig. 5D. Obviously, the AC signal clearly reflects the peak of the captured arterial blood diastolic value. During systole and diastole, the light intensity flux through the arterial blood shows a regular variation and can therefore also be translated into the human heart rate (HR). Monitoring SpO_2_ has vital implications for health and respiratory function, which represents the ratio of oxygen-bound hemoglobin to all bound hemoglobin in the blood. According to the Beer–Lambert law, the SpO_2_ can be determined by the following equation (Eq. 5) in the transmission mode of pulse oximetry [37,38]:

$$\mathrm{Sp}O_{2}=\frac{\varepsilon_{\mathrm{Hb},660}-\varepsilon_{\mathrm{Hb},\;850}R_{\mathrm{OS}}}{\left[\varepsilon_{\mathrm{Hb},660}-\varepsilon_{\mathrm{Hb}O_{2},660}\right]+\left[\varepsilon_{\mathrm{Hb}O_{2},850}-\varepsilon_{\mathrm{Hb},850}\right]R_{\mathrm{OS}}}$$ (5)

where *ε_Hb_* and *ε*_*HbO*_2__ represent the molar extinction coefficients of oxyhemoglobin and deoxyhemoglobin, respectively [39]; *R_os_* is the ratio of the pulsatile (AC) and stationary (DC) signals, which could be derived from the PPG signal and determined by the following equation (Eq. 6):

$$R_{\mathrm{OS}}=\frac{{\mathrm{AC}}_{850}/{\mathrm{DC}}_{850}}{{\mathrm{AC}}_{660}/{\mathrm{DC}}_{660}}$$ (6)

Meanwhile, the HR and SpO_2_ values recorded with the commercial pulse oximetry system (Heal Force A3) are shown in Fig. 5E and F (red curves). The recorded HR and SpO_2_ values by the commercial pulse oximetry system are 72.4 beats per minute and 96.7%, respectively. According to the calculation, HR and SpO_2_ of our device are very close to the commercial system. In future work, we will continue to improve the LED drive circuit and the PPG signal readout circuit to achieve automatic analysis of data signal.

## Conclusions

In conclusion, the photo-induced liquid polarization UV photodetector was realized by integrated polar liquid and a semiconductor. Photogenerated carriers coupled to water molecules and ions have been investigated thoroughly. As a comparison, we confirm that the liquid is not in a conducting state when illuminated, and the current is caused by dynamic a polarization process of the liquid sandwiched between the semiconductors with different Fermi levels. The responsivity and detectivity of transient photo-polarized current in Gr/NaCl (0.5 M)W/N-GaN photodetector reach values of 130.7 mA/W and 2.3 × 10^9^ Jones, respectively. The Gr/NaCl (0.5 M)W/N-GaAs photodetector has a strong optical response in both the Vis and NIR regions. This work integrates polar liquids into heterojunctions, which is expected to provide a new direction for mixed-phase optoelectronic devices by optimizing polar liquid species and additives. We have also achieved a stable non-invasive human oxygen monitoring function based on a polar molecular liquid polarization photodetector. We believe that the innovative physical concept and potential applications will allow this device to be used in the future for IoT applications.

## Materials and Methods

### Device fabrication and measurement

Deionized water, containing a few foreign ions σ < 20 μS/cm, was obtained by a lab water purification system (Heal Force RWD-1). n-Hexane in this work was of analytical grade without further purification. NaCl was purchased form Sinopharm Chemical Reagent Co., Ltd. The epitaxial P-GaN film with Mg concentration ~6×10^19^ cm^−3^, the epitaxial N-GaN film with Si concentration ~6×10^19^ cm^−3^, and 2.2 μm of undoped GaN and 540 nm of N-GaN were grown on a sapphire substrate by metal organic vapor phase epitaxy (HC SemiTek). Au (80 nm) contact was by magnetron sputtering onto one side of substrates successively. The N-type GaAs wafer with doping concentration ~5 × 10^17^ cm^−3^ was thermally evaporated Ti/Au (5 nm/70 nm) in contact with the unpolished back side of GaAs substrate. The GaAs surface needs to be passivated with hydrochloric acid and hydrogen peroxide before it is used [40]. The GaN substrate was ultrasonically cleaned for 10 min. Subsequently, a 150-μm-thick square polyimide tape with a 0.3 mm× 0.5 mm window was transferred onto P-GaN or N-GaN. The graphene was synthesized by the CVD method according to our previous work [19]. Utilizing polyethylene terephthalate (PET) (130 μm) as the support film, the graphene was transferred to the PET surface by the wet transfer method [41,42]. Finally, the PET/Gr was transferred to a window insulating layer made by the PI tape.

### Pulse oximetry measurements

In the pulse oximetry sensor test system, a Gr/NaCl (0.5 M)W/N-GaAs photodetector was used as the receiver. Commercial red (660 nm, 1 W) and NIR (850 nm, 1 W) LEDs were used as light sources. The fingertip of author C. Liu's middle finger was pressed and fixed on a support table with a hole. The LED placed directly above the finger emits incident light and penetrates the tissue, which is detected by a photodetector. The light signal is converted into an electrical signal and further amplified by a low-pass filter. The output signal was recorded by a high-precision 6514 source meter and obtained as HR, SpO_2_, and *R_os_* using the formula in the previous section. All tests were performed under dark conditions to avoid the interference. Since the PPG signal was tested non-invasively on the authors (informed consent), ethical approval was not required.

### Characterization analysis

The *I*–*V* curves were measured by a Keithley 2400 source meter and *I*–*T* curves were carried out by a Keithley 6514 source meter and a DMM6500 multimeter. 1/*f* noise curves were measured by a semiconductor analyzer (PDA, FS-Pro). Photo-current test was measured by a detecting system consisting of a Xe lamp (SOFN Instruments Co., Ltd. 7ILX500C) and a monochromator (SOFN Instruments Co., Ltd. 7ISW15). Raman spectra were measured by a Renishaw system with an excitation laser of 532 nm. Power density of different wavelengths was detected by an S120 VC optical power meter by THOR-LABSU. SEM of graphene/PET was performed by the Zeiss Auriga 40 system. UV–Vis absorption spectra of GaN were recorded by a UV–Vis spectrophotometer (NatureSci Technologies Corporati Lambda950) in the range of 300 to 1,000 nm.

## Data Availability

Supplementary materials contain additional data. All data needed to evaluate the conclusions in the paper are present in the paper or the Supplementary Materials.

## Supplementary Materials

Supplementary 1 Fig. S1. (A) Photographs of Gr/liquid/P-GaN. Fig. S2. Time-resolved photo-polarized voltage of the Gr/W/N-GaN under 350 nm irradiation with different parallel resistors. Fig. S3. Time-resolved photo-polarized current of the Gr/W/N-GaN with different graphene layers. Fig. S4. Time-resolved photo-polarized current of the Gr/n-hexane/N-GaN under 350 nm irradiation. Fig. S5. (A) Voltage–time curves of Gr/NaCl (0.5 M)W/N-GaN with different optical power densities under 350 nm illumination. Fig. S6. The relationship of steady photo-polarized current between the responsivity/detectivity and the incident light power density. Fig. S7. Time-resolved photo-polarized current of the Gr/NaCl (0.5 M)W/N-GaN photodetector with different solution thicknesses under 350 nm irradiation and zero voltage bias. Fig. S8. Photo-polarized current response of Gr/NaCl (0.5 M)W/N-GaN under 350 nm irradiation and zero voltage bias. Fig. S9. SEM image of the graphene/PET (A) before and (B) after repeated measuring. Fig. S10. Responsivity of transient photo-polarized current in (A) Gr/NaCl (0.5 M)W/N-GaN and (C) Gr/NaCl (0.5 M)W/N-GaAs as a function of wavelength under zero bias; corresponding optical power densities are (B) and (D), respectively. Fig. S11. The UV–Vis absorption spectra of N-GaN. Fig. S12. Photograph of the PPG test procedure.
