# Supplementary material for: Self-Driven Photo-Polarized Water Molecule-Triggered Graphene-Based Photodetector
Source: Research (Wash D C). 2023 Jul 31;6:0202. doi: 10.34133/research.0202 (PMC10389694; doi:10.34133/research.0202)
Supplement: Supplementary 1 — Fig. S1. (A) Photographs of Gr/liquid/P-GaN. Fig. S2. Time-resolved photo-polarized voltage of the Gr/W/N-GaN under 350 nm irradiation with different parallel resistors. Fig. S3. Time-resolved photo-polarized current of the Gr/W/N-GaN with different graphene layers. Fig. S4. Time-resolved photo-polarized current of the Gr/n-hexane/N-GaN under 350 nm irradiation. Fig. S5. (A) Voltage–time curves of Gr/NaCl (0.5 M)W/N-GaN with different optical power densities under 350 nm illumination. Fig. S6. The relationship of steady photo-polarized current between the responsivity/detectivity and the incident light power density. Fig. S7. Time-resolved photo-polarized current of the Gr/NaCl (0.5 M)W/N-GaN photodetector with different solution thicknesses under 350 nm irradiation and zero voltage bias. Fig. S8. Photo-polarized current response of Gr/NaCl (0.5 M)W/N-GaN under 350 nm irradiation and zero voltage bias. Fig. S9. SEM image of the graphene/PET (A) before and (B) after repeated measuring. Fig. S10. Responsivity of transient photo-polarized current in (A) Gr/NaCl (0.5 M)W/N-GaN and (C) Gr/NaCl (0.5 M)W/N-GaAs as a function of wavelength under zero bias; corresponding optical power densities are (B) and (D), respectively. Fig. S11. The UV–Vis absorption spectra of N-GaN. Fig. S12. Photograph of the PPG test procedure. [file research.0202.f1.docx]

**Self-driven photo-polarized water molecule triggered graphene-based photodetector**

***Shisheng Lin^1,2,3*^, Chang Liu^1^, Xin Chen^1^, Yi Zhang^4^, Hongtao Lin^1^, Xutao Yu^1^, Yujiao Bo^1^ and Yanghua Lu^2,5^***

**^1^**College of Information Science and Electronic Engineering, Zhejiang University, Hangzhou, 310027, P. R. China.

**^2^**Hangzhou Gelanfeng Technology Co. Ltd, Hangzhou, 310051, P. R. China.

^3^State Key Laboratory of Modern Optical Instrumentation, Zhejiang University, Hangzhou, 310027, P. R. China.

^4^Key Laboratory of Wide Bandgap Semiconductor Materials and Devices, HC Semitek Corporation, Yiwu, 322009, P. R. China.

^5^Smart Materials for Architecture Research Lab, Innovation Center of Yangtze River Delta, Zhejiang University, Jiaxing, 314100, P. R. China.

Email: [shishenglin@zju.edu.cn](mailto:shishenglin@zju.edu.cn)

^*^Corresponding author.


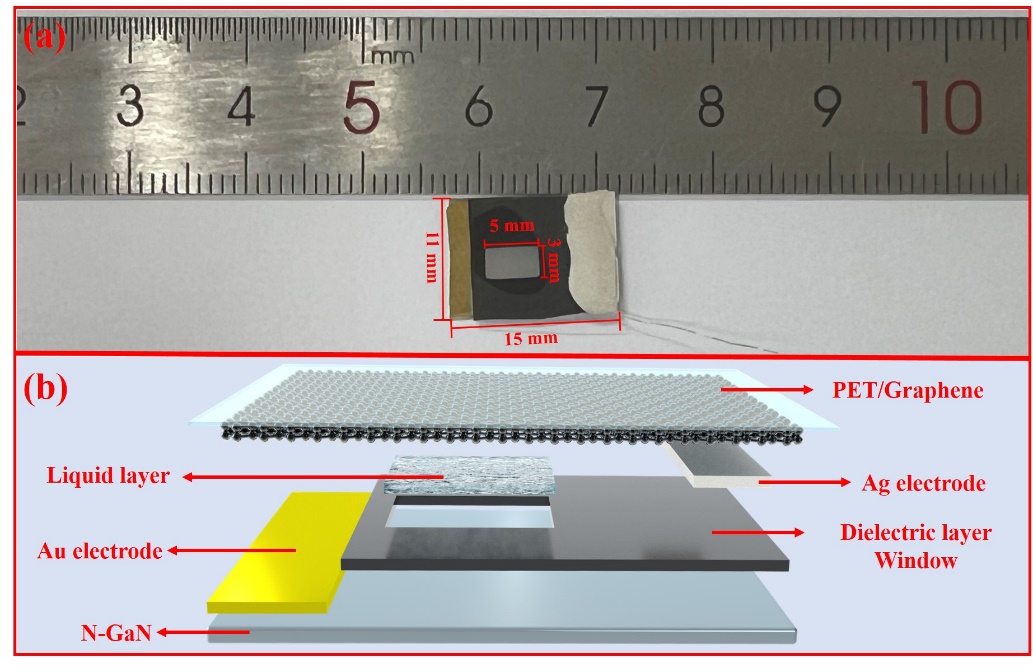


Figure S1. (a) Photographs of Gr/liquid/P-GaN. (b) Scheme of the Gr/liquid/N-GaN with sandwich structure.


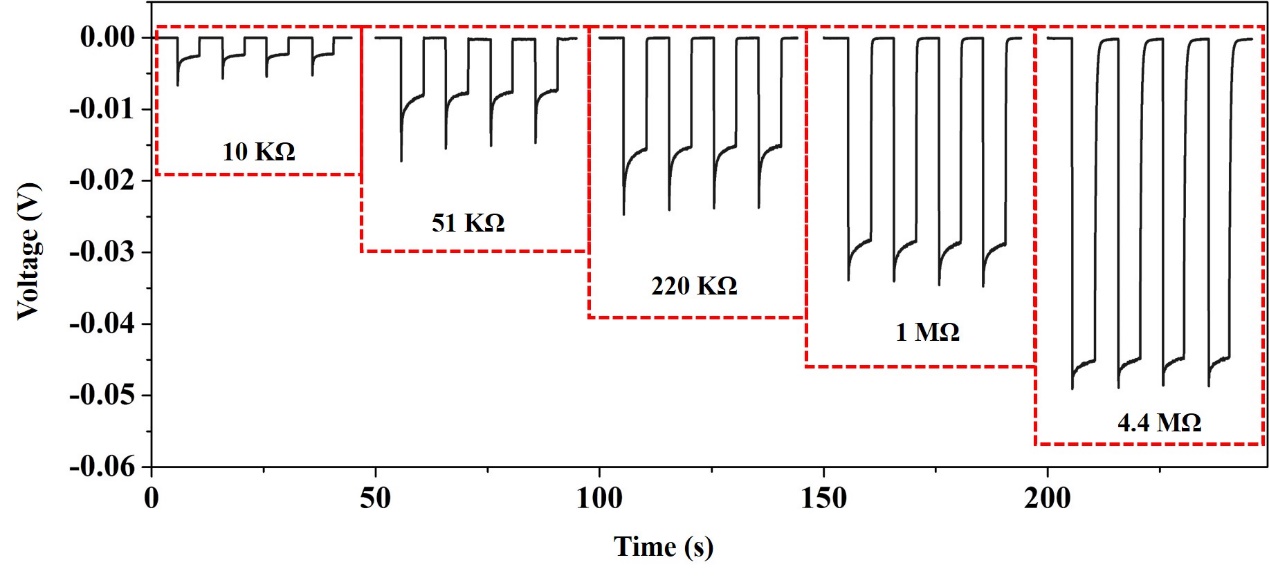


Figure S2. Time-resolved photo-polarized voltage of the Gr/W/N-GaN under 350 nm irradiation with different parallel resistors.


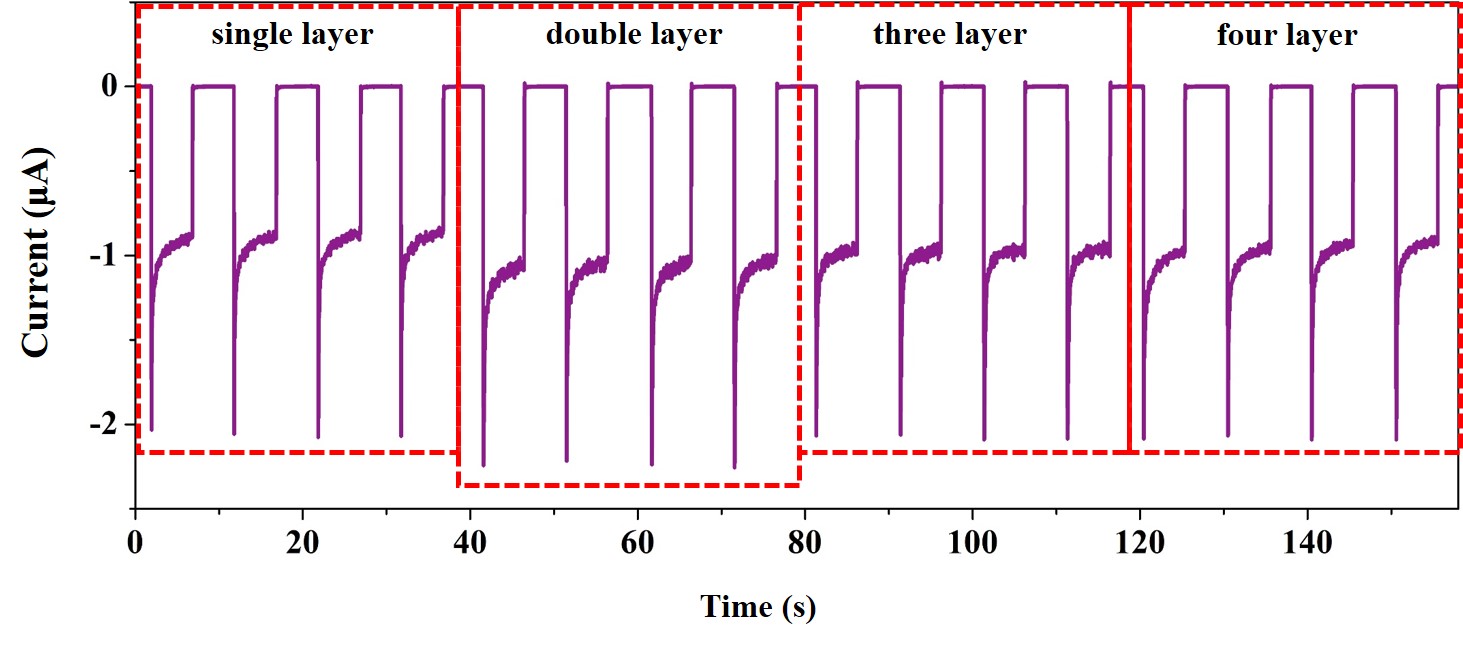


Figure S3. Time-resolved photo-polarized current of the Gr/W/N-GaN with different graphene layers.


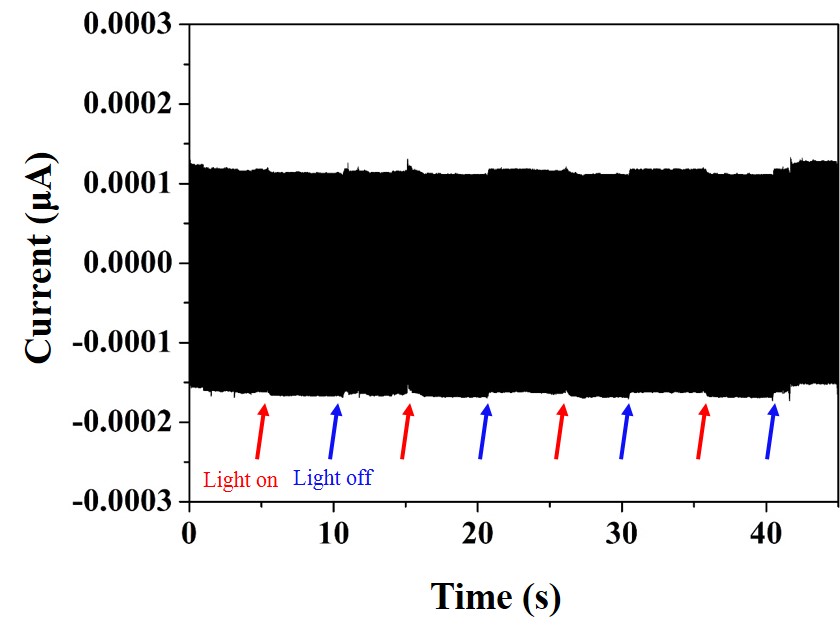


Figure S4. Time-resolved photo-polarized current of the Gr/n-hexane/N-GaN under 350 nm irradiation.


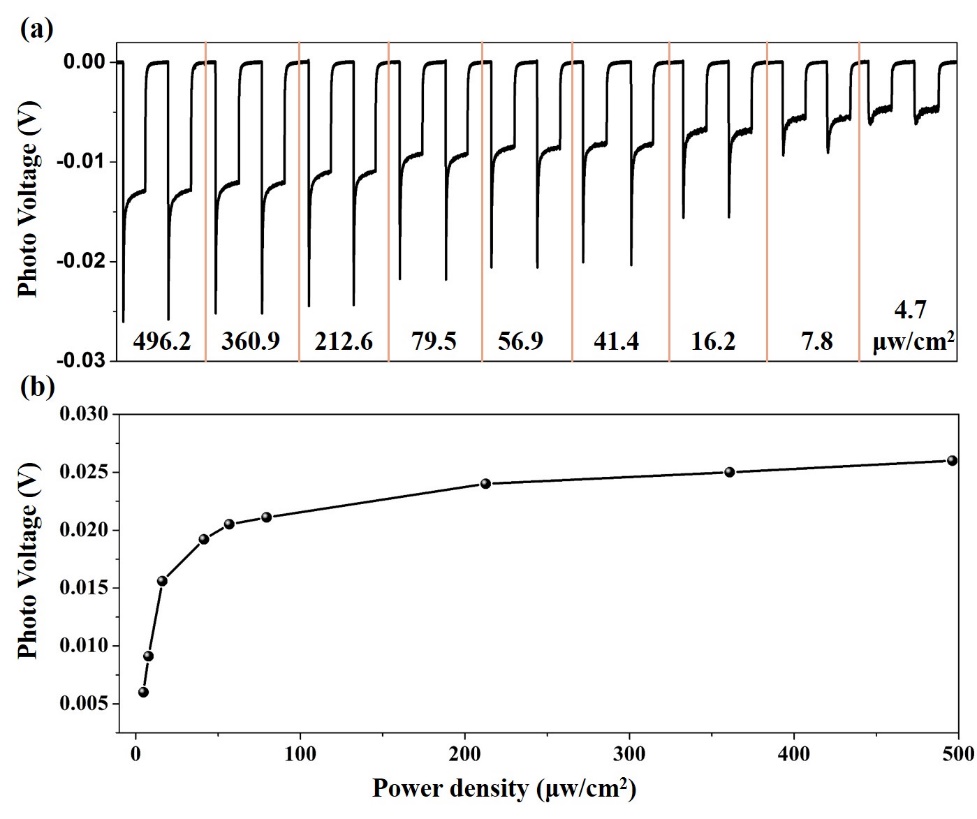


Figure S5. (a) Voltage-time curves of Gr/NaCl (0.5M)/N-GaN with different optical power density under 350 nm illumination. (b) Photo voltage as the function of different optical power density.


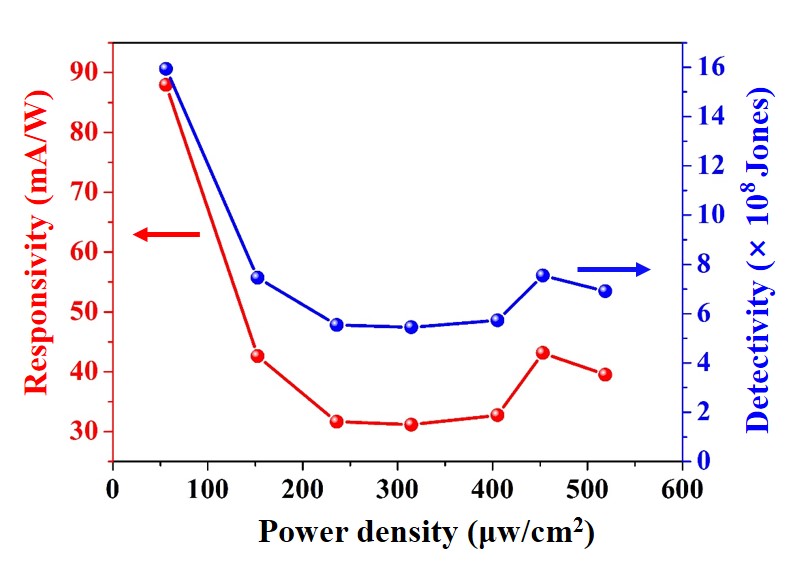


Figure S6. The relationship of steady photo-polarized current between the responsivity/detectivity and the incident light power density.


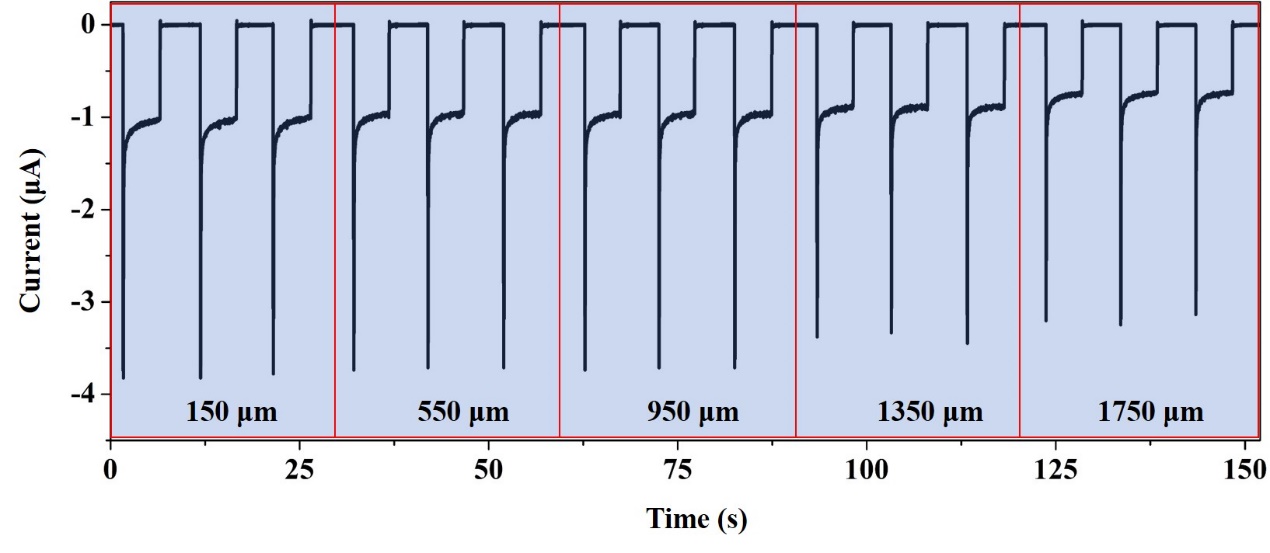


Figure S7. Time-resolved photo-polarized current of the Gr/NaCl (0.5 M)/N-GaN photodetector with different solution thickness under 350 nm irradiation and zero voltage bias.


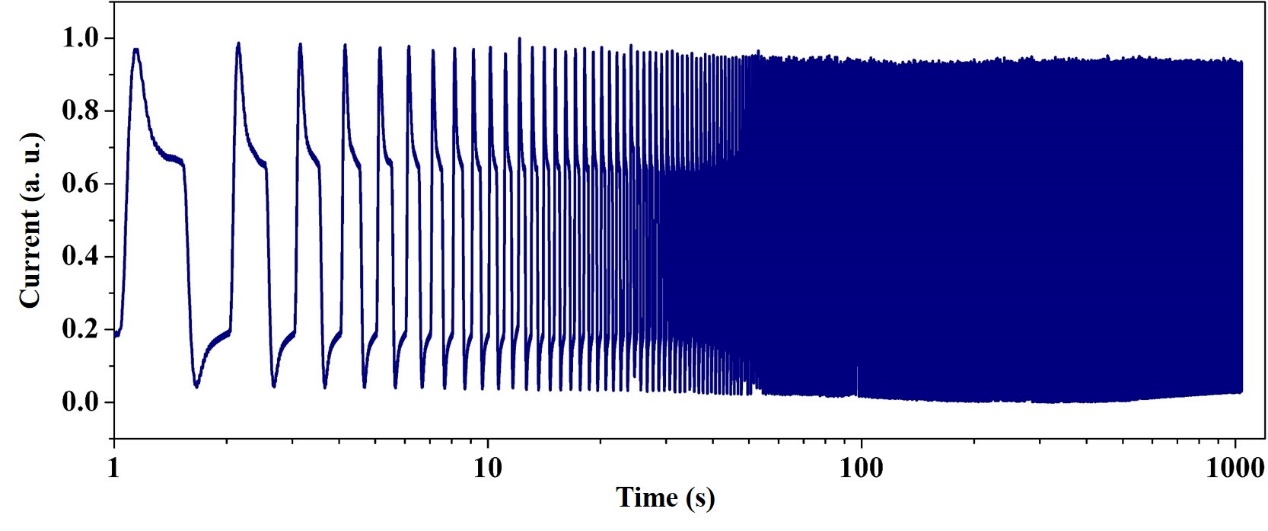


Figure S8. Photo-polarized current response of Gr/NaCl (0.5 M)/N-GaN the under 350 nm irradiation and zero voltage bias.


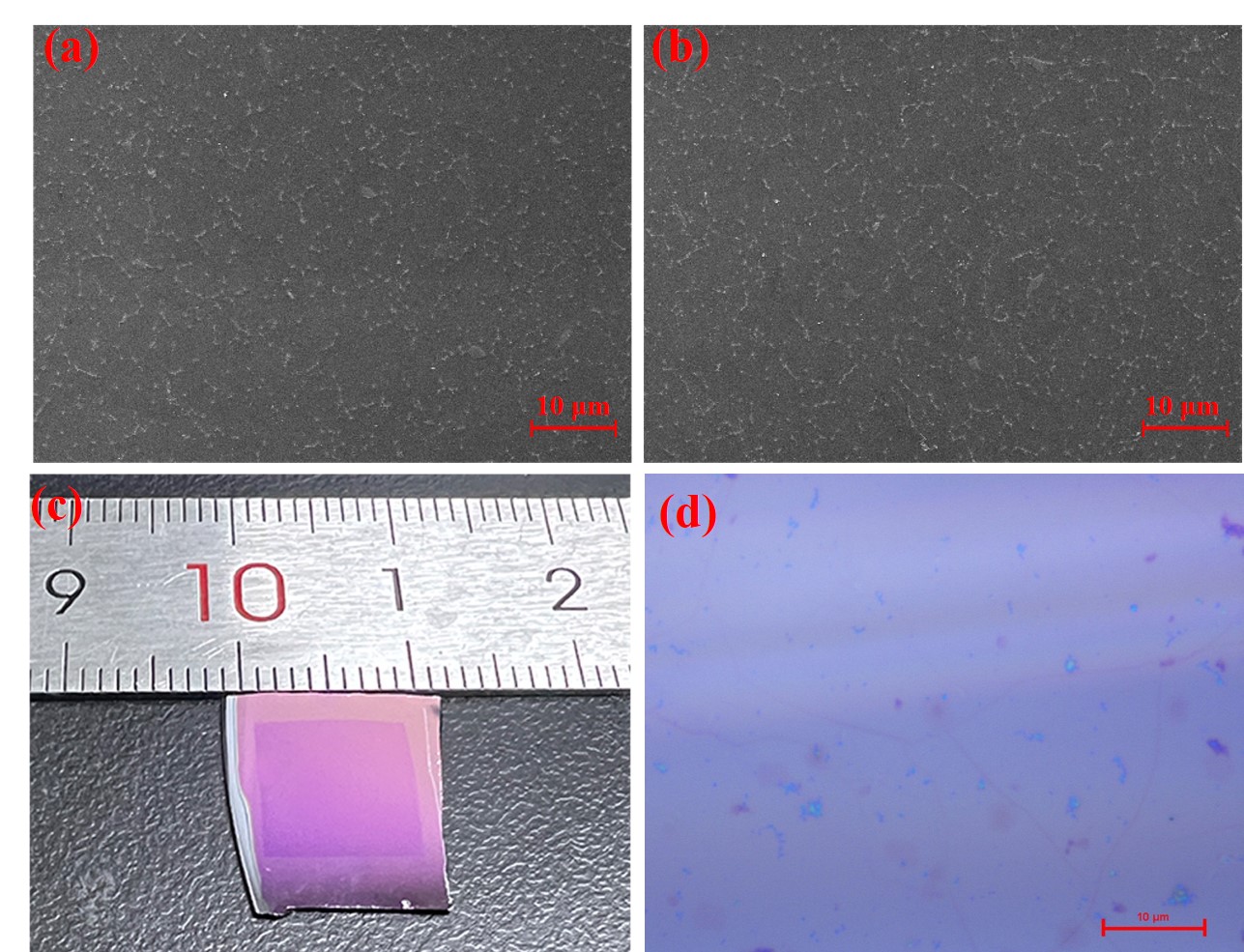


Figure S9. SEM image of the graphene/PET (a) before and (b) after repeatedly measuring. (c) optical photos and (d) optical microscopy image of bi-graphene.


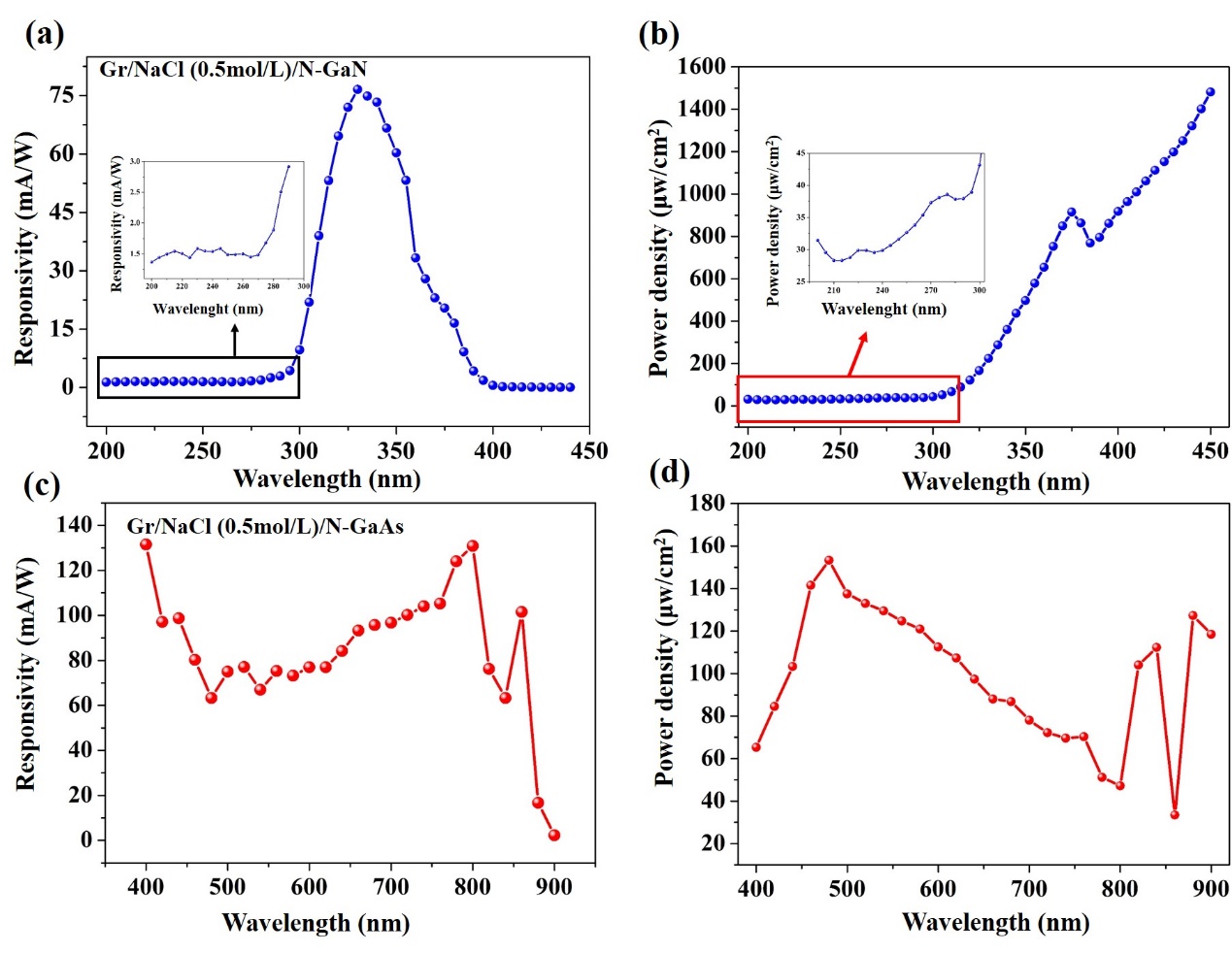


Figure S10. Responsivity of transient photo-polarized current in (a) Gr/NaCl (0.5 M)/N-GaN and (c) Gr/NaCl (0.5 M)/N-GaAs as a function of wavelength under zero bias, corresponding optical power densities are (b) and (d), respectively.


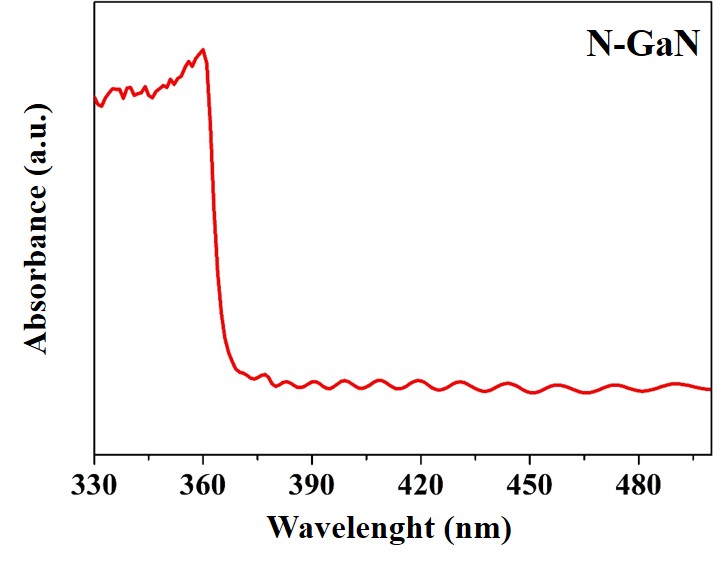


Figure S11. The UV-Vis absorption spectra of N-GaN.


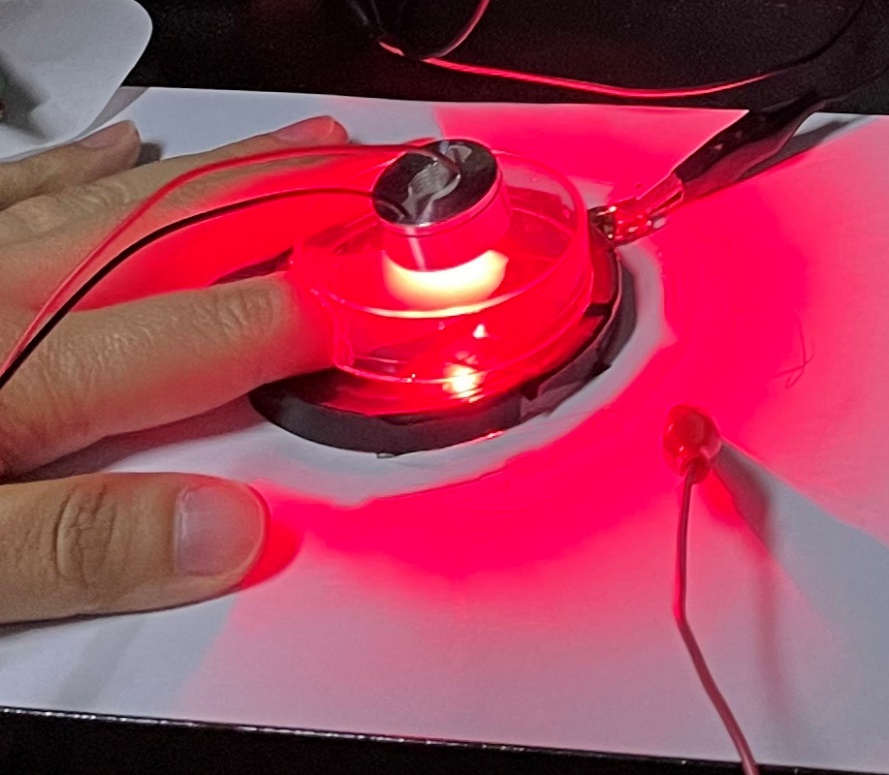


Figure S12. Photograph of PPG test procedure.
